# DNA methylation alone does not cause most cell-type selective transcription factor binding

**DOI:** 10.1186/1756-8935-6-S1-P103

**Published:** 2013-03-18

**Authors:** Matthew T Maurano, Hao Wang, Anthony Shafer, Sam John, John A Stamatoyannopoulos

**Affiliations:** 1 Departments of Genome Sciences, Division of Oncology, University of Washington, Seattle, WA, 98195, USA; 2 Medicine, Division of Oncology, University of Washington, Seattle, WA, 98195, USA

## Background

DNA methylation at vertebrate promoters is associated with the repression of gene expression and is required for mammalian development [1]. The master genome regulator and transcription factor CTCF canonically exhibits methylation-sensitive binding *in vitro*, and its *in vivo* occupancy across cell types anticorrelates with methylation at 41 % of sites genome-wide [2]. However, the genome harbors hundreds of thousands of CTCF recognition sequences, the majority of which are unbound in any cell type. Although most unbound recognition sequences harbor methylation in a given cell type, the causal role of DNA methylation in the abrogation of transcription factor binding at these potential binding sites is largely unknown [3,4].

## Materials and methods

Here we perform genome-wide occupancy profiling after both stable genetic and transient chemical inhibition of DNA methyltransferases in HCT116 cells lacking functional DNMT1 and DNMT3b and K562 erythroleukemia cells treated with 5-aza-2-deoxycytidine.

## Results

We show that the vast majority of susceptible binding sites remain unoccupied upon depletion of DNA methylation. Stable loss of methylation in HCT116 cells does result in a minor increase of several thousand binding sites, largely corresponding to selective reactivation of binding sites from other cell types, especially from other malignant cell lines. Chemical inhibition in K562 cells results in a smaller but reproducible set of several hundred reactivated sites, the majority of which are corroborated by the genetic data. The narrow extent of reactivation implies that the majority of transcription factor recognition sequences are not competent for binding even in the absence of methylation, and that methylation accounts for only a modest fraction of cell-selective binding. These results offer new insight into the establishment of the global regulatory landscape, and offer an important perspective on the interpretation of disease- and trait-associated methylation differences in humans.
